# Case Report: Exchange transfusion for hemolytic anemia and acute kidney injury associated with hepatic arterial infusion chemotherapy with raltitrexed and oxaliplatin in a patient with hepatocellular carcinoma

**DOI:** 10.3389/fmed.2026.1860518

**Published:** 2026-06-18

**Authors:** Caili Zhang, Yajuan Yan, Xiaojian Li, Yong Dong

**Affiliations:** Department of Blood Transfusion, The Affiliated Hospital of Qingdao University, Qingdao, Shandong, China

**Keywords:** acute kidney injury, exchange transfusion, hemolytic anemia, hepatic arterial infusion chemotherapy with raltitrexed plus oxaliplatin, hepatocellular carcinoma

## Abstract

As a highly promising treatment for advanced hepatocellular carcinoma, hepatic arterial infusion chemotherapy with raltitrexed plus oxaliplatin is gaining increasing clinical acceptance. However, in patients with colorectal cancer, treatment with oxaliplatin is associated with severe adverse reactions, such as hemolytic anemia and acute kidney injury. The potential occurrence of such adverse reactions in patients with hepatocellular carcinoma and the corresponding management strategies should be investigated. In this report, we present a case of severe hemolytic anemia and acute kidney injury in a patient with hepatocellular carcinoma after hepatic arterial infusion chemotherapy with raltitrexed plus oxaliplatin therapy, which was suspected to be oxaliplatin-related. The patient achieved complete clinical resolution through two personalized sessions of exchange transfusion, supplemented with artificial liver support therapy.

## 1 Introduction

This case report was approved by the Medical Ethics Committee of the Affiliated Hospital of Qingdao University (Approval No. QYFYWZLL50042). Written informed consent was obtained from the patient. Liver cancer is a leading cause of cancer-related mortality and poses a serious threat to public health and safety worldwide (1). Hepatic arterial infusion chemotherapy (HAIC) significantly extends overall survival in patients diagnosed with advanced, unresectable hepatocellular carcinoma (HCC) (2, 3). The use of raltitrexed and oxaliplatin (RALOX) in HAIC presents a novel treatment strategy for HCC. This regimen appears to demonstrate superior efficacy in reducing infusion time and improving patient tolerance compared to the conventional therapy, such as the combination of 5-fluorouracil and oxaliplatin. However, this chemotherapy protocol is inevitably associated with treatment-related toxicities such as abdominal pain, diarrhea, fever, transaminase elevation, and thrombocytopenia (4, 5).

Oxaliplatin is a third-generation platinum-based antineoplastic agent that inhibits DNA replication and transcription, thereby inducing tumor cell apoptosis. It is primarily used in the treatment of colorectal cancer, gastric cancer, and pancreatic cancer (6). Common adverse reactions to this drug include fever, gastrointestinal bleeding, myelosuppression, and peripheral neuropathy (7). Previous case reports have demonstrated that oxaliplatin, when used in the treatment of patients with colorectal cancer, can induce both immune and non-immune hemolytic anemia (HA), Evans syndrome, and acute renal failure, posing a serious threat to patient survival (8, 9). The discontinuation of the drug, corticosteroid therapy, or the combination with therapeutic plasma exchange can effectively ameliorate hemolytic symptoms and mitigate tissue and organ damage. To our knowledge, no previous cases of oxaliplatin-related HA and acute kidney injury (AKI) have been documented in patients with HCC.

This is the first documented case of HA and AKI in a patient with HCC following RALOX-HAIC, and these complications were likely induced by oxaliplatin. In this case, a tailored exchange transfusion (ET) strategy was developed based on the evolving clinical condition of the patient, resulting in satisfactory therapeutic outcomes over a brief period of time. The scope of this report is focused on the clinical presentation, dynamic evolution of laboratory parameters, and critical decision-making process, with the aim of providing insights for recognizing and managing this rare but serious chemotherapy-associated complication.

## 2 Case report

The patient was a 55-year-old male initially diagnosed with HCC in 2018 and treated with microwave ablation therapy and transarterial chemoembolization. Subsequently, he underwent RALOX-HAIC therapy at the Affiliated Hospital of Qingdao University (Qingdao, China) on March 17, 2025, for advanced HCC complicated by portal vein tumor thrombosis. Prior to this admission, the patient had not received any oxaliplatin-containing treatment regimen. The procedure, which proceeded smoothly, consisted of hepatic artery catheterization subsequent to angiography that demonstrated multifocal tumor staining. The intervention was followed by an uneventful pump-mediated infusion of the drug regimen [oxaliplatin 150 mg (Qilu Pharmaceutical (Hainan) Co., Ltd., lot FB1Y4018) plus raltitrexed 4 mg (Nanjing Chia-tai Tianqing Pharmaceutical Co., Ltd., lot 2,409,052)]. The sole adverse effect reported by the patient was mild nausea. However, the patient experienced a sudden onset of high fever accompanied by chills, with the body temperature reaching 40 °C. Intermittent symptomatic management was administered, including antipyretics (arginine aspirin), corticosteroids (dexamethasone), and physical cooling methods, supplemented with intravenous fluid resuscitation and empirical antibiotic therapy.

On March 20, 2025, the patient experienced exacerbated abdominal pain, widespread jaundice, and excreted dark, tea-colored urine via the catheter. Laboratory results indicated a substantial increase in hemolytic markers. Based on the patient’s clinical manifestations and laboratory findings, other etiologies of hemolysis were preliminarily excluded. The patient had no history suggestive of glucose-6-phosphate dehydrogenase (G6PD) deficiency, and hemolysis continued to progress despite anti-infective therapy, with negative blood and urine cultures. Although ADAMTS13 activity and its antibody testing were not performed, the absence of overt bleeding or neurological symptoms largely ruled out thrombotic thrombocytopenic purpura. A negative direct antiglobulin test (DAT) largely ruled out autoimmune HA. Given the timing of the onset of hemolysis in relation to RALOX-HAIC therapy, oxaliplatin-associated HA was strongly suspected. Accordingly, ET was immediately initiated.

During the first session of ET, the patient presented with hypotension (92/58 mmHg) and possible cardiac reserve impairment. Given the potential high risk associated with rapid and large-volume ET, it was decided to exchange approximately two-thirds of the total blood volume (TBV) per session, equivalent to about 3,700 mL (TBV: body weight 80 kg × 70 mL/kg = 5,600 mL), with a 110% fluid balance (i.e., the volume of infused fluid was 10% greater than the volume of removed blood). The primary objectives of this ET were to rapidly restore the hematocrit, remove damaged and potentially sensitized red blood cells (RBCs), and correct the patient’s anemic symptoms. Therefore, the ratio of RBC-to-plasma in the replacement fluid was determined primarily based on the calculated RBC volume. Accordingly, the estimated RBC volume for the first ET was 1,640.8 mL (preoperative hematocrit 29.3% × TBV 5,600 mL), and the estimated plasma volume was 2,059.2 mL. A single session of ET was expected to remove 50% of the patient’s native RBCs. Considering both the relief of the patient’s symptoms and the conservation of valuable blood resources, the estimated RBC volume for the second ET was 820.4 mL (preoperative hematocrit 29.3% × TBV 5,600 mL × 50%), with an estimated plasma volume of 2,879.6 mL. Given that each unit of stored blood components has a fixed volume specification and the actual volume is not highly precise (labeled volume ±10%), the actual blood volume used during the procedure may deviate slightly from the estimated volume. In addition, serum calcium levels were dynamically monitored before, during, and after the ET, enabling timely intervention for citrate-induced hypocalcemia.

On March 20, 2025, the first ET was carried out. The procedure actually involved the administration of 1,600 mL of leukocyte-reduced RBCs, 2,100 mL of fresh frozen plasma, and 300 mL of normal saline, with a total of 3,206 mL of fluid removed. Before treatment, the patient had a low ionized calcium level (0.97 mmol/L). Therefore, 20 mL of calcium gluconate solution (prepared by adding 2 mL of 10% calcium gluconate injection to 18 mL of glucose solution) was prophylactically administered as a continuous infusion via a syringe pump at the start of the procedure. When perioral and limb numbness developed during the ET, an additional dose of the same solution was given, leading to resolution of hypocalcemic symptoms. On the evening of the procedure and the following day, the patient’s hemoglobin (Hb) levels increased to more than 70 g/L, accompanied by a marked decrease in indirect bilirubin and a gradual normalization of serum potassium levels. These findings indicated the cessation of ongoing hemolysis. On March 21, 2025, the clinical team administered one session of artificial liver support therapy to the patient. Although serum indirect bilirubin levels continued to decline on March 22 and 23, 2025, Hb levels also decreased, suggesting that some RBCs were still being destroyed. Subsequently, the second procedure was performed on March 23, 2025. This session used 600 mL of leukocyte-reduced RBCs, 2,410 mL of fresh frozen plasma, and 750 mL of normal saline, with a total exchange volume of 3,668 mL. During this treatment session, the patient’s ionized calcium concentration decreased from 1.14 to 1.09 mmol/L, and no symptoms of hypocalcemia were observed; thus, no calcium gluconate solution was given. Following this treatment, laboratory markers of hemolysis showed a continual positive trend, indicating effective management of the condition. The patient’s serum lactate dehydrogenase levels reverted to pre-treatment levels following two sessions of ET, further validating the efficacy of this intervention in managing hemolysis. Notably, despite the absence of platelet transfusions, the decline in platelet count was halted after the first ET session and began to recover after the second session (Table 1). This observation indicates that ET effectively mitigated the ongoing consumption and destruction of platelets.

**Table 1:** Hemolytic parameters before and after exchange transfusion (ET).

Timepoint	Pre-chemotherapy	3.20^a^	3.20^b^	3.21	3.22	3.23^c^	3.23^d^	3.24	3.25	3.31	Reference range
RBC (×10^12^/L)	3.24	1.93	2.18	2.19	2.12	1.82	2.30	2.32	2.43	2.40	4.3–5.8
Hb (g/L)	95	67	71	72	66	58	70	71	73	75	130–175
PLT (×10^9^/L)	178	206	112	104	87	79	62	70	89	100	125–350
K + (mmol/L)	4.09	5.9	5.2	4.1	3.9	4.1	3.5	4.2	3.9	3.9	3.5–5.3
IBIL (μmol/L)	6.1	107.2	81.9	57.9	53.9	43.9	37.5	38.4	35.3	26.0	0–19
LDH (U/L)	334	1,303	/	/	/	/	/	/	/	334	120–250

RBC, red blood cells; Hb, hemoglobin; PLT, platelets; K+, serum potassium; IBIL, indirect bilirubin; LDH, lactate dehydrogenase; ^a^pre-first ET; ^b^post-first ET; ^c^pre-second ET; ^d^post-second ET.

**Figure 1:**
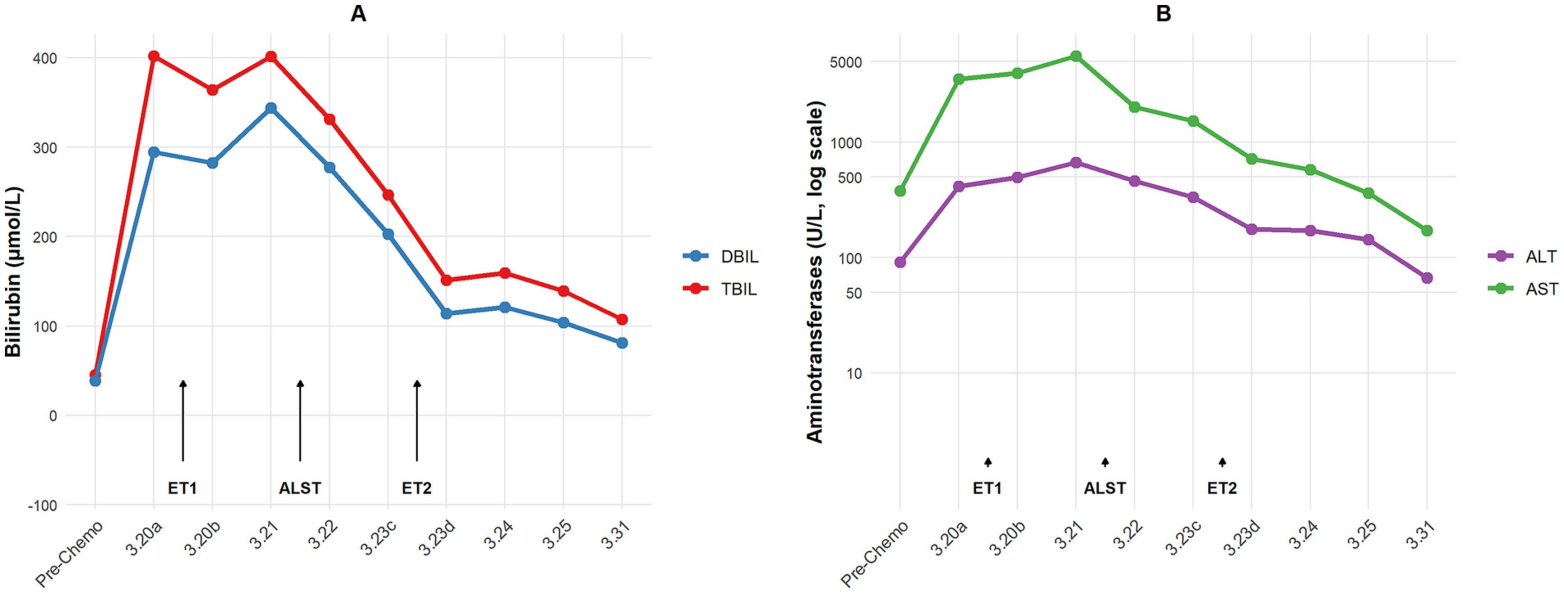
Changes in liver function parameters before and after exchange transfusion (ET). The patient also received artificial liver support therapy (ALST) concurrently. **(A)** Changes in TBIL and DBIL over time. The vertical dashed lines indicate the timing of the first (ET1) and the second (ET2) exchange transfusion. **(B)** Changes in ALT and AST over time (log scale). ^a^pre-first ET; ^b^post-first ET; ^c^pre-second ET; ^d^post-second ET. ALT, alanine aminotransferase; ALST, artificial liver support therapy; AST, aspartate aminotransferase; DBIL, direct bilirubin; ET, exchange transfusion; TBIL, total bilirubin. The *y*-axis for aminotransferases is presented on a logarithmic scale to accommodate the wide range of values.

The significant reduction and subsequent stabilization of hepatic parameters post-treatment, including total bilirubin, direct bilirubin, alanine aminotransferase, and aspartate aminotransferase, along with preserved synthetic function (evidenced by albumin and prealbumin levels), indicated that liver injury did not progress (Figures 1A,B). Prior to treatment, the patient’s serum creatinine levels increased from 50.1 μmol/L to 91.7 μmol/L. Urinalysis revealed red-colored urine with 2 + occult blood, 3 + proteinuria, 3 + bilirubin, and 3 + urobilinogen. Microscopic examination showed granular casts and a mixed population of erythrocytes, findings consistent with AKI. After two sessions of ET, the patient’s urine appeared yellow with only 1 + proteinuria. Serum creatinine and blood urea nitrogen concentrations decreased, indicating an improvement in renal function. However, six weeks later, the patient’s serum creatinine, blood urea nitrogen, cystatin C, and β_2_-microglobulin levels began to rise, accompanied by a significant decline in the glomerular filtration rate (Table 2).

**Table 2:** Renal parameters before and after exchange transfusion (ET).

Timepoint	Pre-Chemotherapy	3.20^a^	3.20^b^	3.24	5.1	5.7	5.14	5.27	References range
SCr (μmol/L)	50.1	91.7	109.4	96.8	78.2	100.4	147.7	161.7	57–97
BUN(mmol/L)	3.2	10.4	14.7	18.0	8.2	13.6	15.6	17.0	_
cystatin C(mg/L)	1.06	/	/	/	2.41	3.20	3.07	3.01	0.51–1.09
β_2_-microglobulin(mg/L)	2.25	/	/	/	5.74	7.57	9.15	9.82	1.0–2.3
GFR (mL/min/1.73 m2)	115.87	/	/	/	97.15	71.82	45.03	40.36	_

BUN, blood urea nitrogen; GFR, glomerular filtration rate; SCr, serum creatinine; ^a^pre-first ET; ^b^post-first ET.

During the entire treatment, no abnormal fluctuations in blood pressure or heart rate were observed, and no ET-related adverse transfusion reactions (such as allergy, infection, or hemolysis) occurred, apart from a promptly managed episode of citrate-induced hypocalcemia.

## 3 Discussion

As a novel treatment regimen for advanced HCC, there are limited real-world clinical data for RALOX-HAIC. Consequently, no cases of HA associated with this therapy have been reported thus far. Raltitrexed, an alternative to 5-fluorouracil, is associated with minimal side effects. Nevertheless, the occurrence of oxaliplatin-related HA has been confirmed in multiple studies (10–12). Previous studies have demonstrated that anti-oxaliplatin antibodies are frequently detectable in the serum of patients receiving repeated oxaliplatin-based chemotherapy, and these patients often have a positive DAT on their erythrocytes. These findings confirm that the formation of drug-dependent antibodies is one of the mechanisms underlying oxaliplatin-associated HA (13). However, oxaliplatin-associated HA may also be related to the non-immunological protein adsorption (NIPA) pathway. Oxaliplatin can directly bind to the erythrocyte membrane, alter its surface properties, and induce non-specific adsorption of plasma proteins (e.g., albumin, immunoglobulins, complement components), ultimately leading to hemolysis (14, 15). Arndt et al. demonstrated that oxaliplatin-sensitized erythrocytes reacted with normal human serum and anti-globulin reagents, providing evidence for the NIPA pathway (16). In this case, due to the urgent clinical situation at the time, we were unable to perform timely testing for anti-oxaliplatin antibodies in the patient’s serum, which represents a major limitation of this report. Nevertheless, the patient had no prior exposure to oxaliplatin, and other etiologies of hemolysis were excluded. Following two sessions of ET, marked improvement was observed in hemolytic symptoms as well as liver and renal function. These findings strongly suggest that the hemolysis in this case was most likely oxaliplatin-related and may have involved the NIPA pathway. Although the DAT for both anti-immunoglobulin G and anti-C3d was negative, this does not rule out the NIPA pathway, especially in the setting of severe hemolysis with rapid complement consumption (17). Negative DAT results can be attributed to either a low quantity of surface-bound protein on erythrocytes or extensive destruction of abnormal cells, which may reduce the target cell population to below the assay’s detection threshold. However, other potential mechanisms cannot be completely excluded, such as the patient’s glutathione S-transferase gene polymorphism or underlying liver dysfunction, which could lead to the accumulation of oxaliplatin metabolites, directly damaging the erythrocyte membrane or triggering oxidative stress, thereby resulting in non-immunological hemolysis (18, 19). Given the above conditions, acute hemolysis is highly likely to develop, even upon the patient’s first exposure to oxaliplatin.

Currently, the management of oxaliplatin-related HA typically involves combination therapy, which may include high-dose corticosteroids, hemodialysis, therapeutic plasma exchange, component blood transfusion, or intravenous immunoglobulin infusion (9, 13). In this case, two sessions of ET were performed, with one session of artificial liver support therapy (the dual plasma molecular adsorption system, DPMAS) administered between the two exchange sessions. This ET-DPMAS-ET sequential regimen was designed to achieve rapid and effective correction of the patient’s anemic and hypoxic symptoms, as well as comprehensive, multi-modal clearance of pathogenic substances from the serum. Based on the patient’s preoperative hematocrit, TBV, and the primary therapeutic goal, we determined the volumes of RBCs and plasma for the first ET. This initial ET aimed to rapidly replenish normally functioning RBCs, while removing damaged pathological RBCs, free hemoglobin, free oxaliplatin, and its albumin-bound macromolecular complexes, along with other pathogenic substances. Subsequently, DPMAS was performed to selectively adsorb medium-molecular-weight substances in the patient’s serum, such as bilirubin and potentially elevated pro-inflammatory cytokines. Considering that a single ET session approximately removes 50% of the patient’s native RBCs, and accounting for the potential continued release of hemolytic byproducts, we estimated the blood component volumes required for the second exchange session. The results showed that RBC count and Hb levels ceased to decline by the day following the first ET session. Concurrently, K^+^ normalized and indirect bilirubin concentrations continued to decline, with marked improvement in the patient’s anemic symptoms. Following two personalized ET sessions and one session of DPMAS, hemolysis completely resolved, liver function recovered significantly, and efficient utilization of blood products was achieved. The structured decision-making parameters of our ET protocol are presented in Supplementary Table 1. Compared with therapeutic plasma exchange alone, the advantage of ET in this case was that it rapidly increased hematocrit, improved anemic symptoms, and removed sensitized RBCs, thereby preventing further destruction of the patient’s own erythrocytes while also eliminating various harmful substances from the plasma. Although the artificial liver support therapy administered between the two ET sessions was similarly effective at clearing serum toxins, its role in preventing the destruction of the RBCs and correcting anemia was very limited. This further indicates that timely ET was essential in this case. According to a previous report, two sessions of ET led to resolution of hemolysis within 3 days and the normalization of platelet count (100 × 10^9^/L) by day 21 (15). Notably, in this case, we observed more rapid clinical resolution and platelet recovery; specifically, clinical resolution was achieved immediately after the first exchange, and platelet recovery to 100 × 10^9^/L occurred by day 8. This accelerated response underscores the superior efficiency of ET for managing this condition compared with therapeutic plasma exchange. The absence of serious adverse events throughout the treatment period indicates the excellent safety profile of this intervention. Furthermore, the patient reported significant improvement in fatigue and dizziness, and both the patient and his family expressed satisfaction with the treatment outcome.

Following the destruction of erythrocytes, free Hb is released into the bloodstream, generating reactive oxygen species such as hydrogen peroxide and superoxide radicals. These reactive oxygen species induce vascular oxidative stress and subsequent tissue damage. ET promotes the recovery of Hb levels and RBCs, effectively removes hemolytic products, and inhibits oxidative stress and inflammation, thereby mitigating organ damage. The patient developed AKI after HA. Although some follow-up data were missing, serial serum creatinine measurements showed a transient improvement in renal function shortly after two sessions of ET. This suggests that ET effectively cleared circulating nephrotoxic substances such as free hemoglobin, thereby alleviating acute tubular injury. However, renal function progressively deteriorated more than 1 month after treatment, indicating that ET did not completely prevent the long-term progression of kidney injury. The reasons for this outcome are multifactorial. Oxaliplatin itself has direct nephrotoxic effects. Its hydrolytic metabolites can trigger DNA damage in renal tubular epithelial cells, leading to tubular necrosis and progressive renal function decline (20). Additionally, systemic hemodynamic alterations and renal vasoconstriction associated with hepatocellular carcinoma and underlying cirrhosis may persist after ET, contributing to insidious renal function deterioration (21). Furthermore, N-acetylcysteine, a potential renoprotective agent that mitigates oxidative stress-induced tubular damage, was not administered in the early phase of hemolysis-associated renal injury (22). This may have also contributed to the unfavorable outcome. Therefore, although ET may provide transient relief of AKI during the acute phase, the patient’s renal function showed chronic progressive deterioration due to multiple factors, including the direct nephrotoxicity of oxaliplatin, the persistent impact of underlying liver disease on renal function, and the lack of comprehensive renoprotective measures.

The diagnostic challenges encountered in this case highlight the importance of a systematic approach to suspected oxaliplatin-related HA. Based on our experience, we propose a practical protocol for similar cases to facilitate a definitive diagnosis. Before initiating any empirical treatment (including ET, artificial liver support, or corticosteroids), blood samples should be collected as follows: (1) Routine laboratory evaluation: Collect blood for complete blood count, liver and renal function tests, and DAT. (2) Antibody testing: Collect at least 5–10 mL of serum. Immediately centrifuge and aliquot, then store at −20 °C or −80 °C for subsequent anti-oxaliplatin antibody testing (13). This archived sample may also be used to test for other antibodies (e.g., anti-ADAMTS13) for differential diagnosis of hemolysis. (3) Enzymatic and genetic testing: Reserve whole blood samples for subsequent analysis (e.g., G6PD deficiency testing and genetic studies) to help exclude other etiologies of hemolysis (23). Collect one ethylenediaminetetraacetic acid-anticoagulated whole blood sample, split it into two aliquots, and store one at 2–8 °C (for use within 7 days) and the other at −20 °C or −80 °C for long-term preservation. At present, no specific laboratory test exists for oxaliplatin-associated non-immunological hemolysis, and the diagnosis is largely based on the exclusion of other identifiable causes of hemolysis. Notably, ET is a life-saving emergency intervention for such patients and should not be delayed while awaiting serological confirmation. The collection and storage of blood samples are intended for retrospective diagnostic analysis, which may guide decisions regarding the use of oxaliplatin in future treatment regimens.

## 4 Conclusion

This case demonstrates the efficacy and safety of personalized ET as a life-saving intervention for RALOX-HAIC-induced HA and AKI. This approach is capable of correcting anemia, controlling the progression of hemolysis, rapidly eliminating harmful substances from the blood, and protecting against organ damage. Nevertheless, it should be noted that while ET may provide short-term renal function improvement, it may not prevent the progression of chronic kidney disease. This observation requires further evaluation and exploration in future studies.

## Data Availability

The datasets presented in this study can be found in online repositories. The names of the repository/repositories and accession number(s) can be found in the article/Supplementary material.
