# Supplementary material for: Case Report: Exchange transfusion for hemolytic anemia and acute kidney injury associated with hepatic arterial infusion chemotherapy with raltitrexed and oxaliplatin in a patient with hepatocellular carcinoma
Source: Front Med (Lausanne). 2026 Jun 18;13:1860518. doi: 10.3389/fmed.2026.1860518 (PMC13322861; doi:10.3389/fmed.2026.1860518)
Supplement: Supplementary file 1 [file Table_1.docx]

**Supplementary Table 1.** Clinical Decision-making Summary for our exchange transfusion (ET) protocol

| Decision Parameter | Our Protocol | Rationale/Evidence Base |
| --- | --- | --- |
| a) Indications for ET initiation | 1) Severe, progressive acute hemolytic anemia (e.g., Hb < 70 g/L, persistently elevated LDH and IBIL).  2) Acute hepatic and renal injury (e.g., elevated TBIL/DBIL, increased SCr, hematuria, proteinuria).  3) Failure of initial medical or supportive therapy. | There is no universally accepted standard for ET in drug-related hemolytic anemia. These indications are based on our institutional experience and reference the American Society for Apheresis (ASFA) guidelines for therapeutic plasma exchange, which list conditions such as autoimmune hemolytic anemia and acute kidney injury. |
| b) TBV calculation formula | TBV (ml) = body weight (kg) × 70 mL/kg | Standard estimation for adult males. Alternative: 65 mL/kg for females or elderly. |
| c) Exchange volume selection | 3,700 mL (approximately two-thirds of TBV) | Given the patient’s hypotension (92/58 mmHg) on admission, a large-volume ET was considered to carry a high risk of hemodynamic instability. Therefore, an exchange volume of approximately two-thirds TBV was selected, which achieves a theoretical clearance of approximately 50% [clearance = 1 - *e*^(-V_exchange/TBV)], while preserving hemodynamic safety.  We emphasize that this value is not an absolute standard but an individualized threshold derived from dynamic risk-benefit assessment. For hemodynamically stable patients, higher exchange volumes (e.g., 0.8–1.0 × TBV) may be considered. |
| d) RBC-to-plasma ratio | Session 1: ~1:1.5 | Calculation: V_RBC1 = preoperative hematocrit × TBV = 29.3% × 5,600 mL= 1,640.8 mL; V_plasma1 = V_exchange - V_RBC1 = 3,700 mL - 1,640.8 mL = 2,059.2 mL.  Goal: Rapidly restore hematocrit and halt hemolysis progression. |
|  | Session 2: ~1:4 | Calculation: V_RBC2 = V_RBC1 × (1 - clearance) = 1640.8 mL × 50% = 820.4 mL; V_plasma2 = V_exchange - V_RBC2 = 3,700 mL - 820.4 mL = 2,879.6 mL.  Goal: Based on reduced hemolysis after session 1, higher plasma proportion was used to enhance clearance of pathogenic plasma components (e.g., bilirubin, free hemoglobin). |
| e) Fluid balance coefficient | 110% (i.e., the volume of infused fluid was 10% greater than the volume of removed blood) | ASFA guidelines recommend individualizing fluid balance based on hemodynamic status,with positive balance (101–120%) for hypotension. |
| f) Hypocalcemia prophylaxis | 1) Routinely measure ionized calcium before ET.  2) If ionized calcium ＜ 1.0 mmol/L, administer prophylactic calcium gluconate (10%, 20 mL) via pump.  3) If hypocalcemia symptoms develop, administer additional calcium gluconate (10%, 20 mL). | Citrate-induced hypocalcemia is a well-recognized complication of ET. ASFA guidelines recommend routine ionized calcium monitoring with preventive calcium supplementation during plasma exchange procedures. |

ASFA, American Society for Apheresis; DBIL, direct bilirubin; Hb, hemoglobin; IBIL, indirect bilirubin; LDH, lactate dehydrogenase; RBC, red blood cells; SCr, serum creatinine; TBIL, total bilirubin; TBV, total blood volume; V_exchange, estimated exchange volume; V_plasma1 and V_RBC1, estimated plasma and RBC volume for the first ET session; V_plasma2 and V_RBC2, estimated plasma and RBC volume for the second ET session.
